# Empyema caused by MRSA ST398 with Atypical Resistance Profile, Spain

**DOI:** 10.3201/eid1701.100307

**Published:** 2011-01

**Authors:** Carmen Lozano, Carmen Aspiroz, Ana Isabel Ezpeleta, Elena Gómez-Sanz, Myriam Zarazaga, Carmen Torres

**Affiliations:** Author affiliations: Universidad de La Rioja, Logroño, Spain (C. Lozano, E. Gómez-Sanz, M. Zarazaga, C. Torres);; Hospital Royo Villanova, Zaragoza, Spain (C. Aspiroz, A.I. Ezpeleta)

**Keywords:** Methicillin-resistant Staphylococcus aureus, empyema, pigs, bacteria, MRSA, ST398, Spain, zoonoses, letter

**To the Editor:** We report a case of empyema caused by methicillin-resistant *Staphylococcus aureus* (MRSA) sequence type ST398 in a 79-year-old man in Spain who had severe chronic obstructive pulmonary disease. In 2009, the patient was hospitalized in the intensive care unit because of decompensation of his chronic obstructive pulmonary disease, profound iliofemoral venous thrombosis, right pneumothorax, and lung carcinoma. Thoracic drainage, support measures, and intravenous levofloxacin were initiated, but no clinical improvement was seen. Purulent exudates from the thoracic drainage tube and of a tracheal aspirate were cultured. MRSA was isolated from both samples and from a nasal swab. Antimicrobial drug therapy was changed from levofloxacin to intravenous linezolid, but the patient’s clinical situation rapidly worsened, and he died of multiorgan failure.

The 3 MRSA isolates were typed (multilocus sequence typing-, *spa*-, staphylococcal cassette chromosome [SCC] *mec*-, and *agr*-typing, in addition to pulsed-field gel electrophoresis [PFGE]), likewise, the antimicrobial drug–resistance phenotypes and genotypes, and virulence genes were determined (*1**,**2*). All 3 MRSA isolates were typed as sequence type (ST) 398, *spa*-type t011, SCC*mec*V, and *agr*I. The 3 isolates had the same resistance phenotype, including to β-lactams, tetracycline, clindamycin (but not erythromycin), ciprofloxacin, and levofloxacin. We confirmed the presence of *mec*A, *tet*M, *tet*K, and *vga*(A) genes by PCR and sequencing; however, PCRs for *lnu*(A), *lnu*(B), *lnu*(C), *lnu*(D), *cfr, vga*(C), *lsa*(B), and *tetL* genes were negative. Primers used for detection of *vga*(A) gene were F-5′-GAAACTCTTATTCGAACYATTCTAGC-3′ and R-5′-GGTTCAATACTCAATCGACTGAG-3′. Specific amino acid changes in the quinolone-determining region of GyrA and ParC proteins (S84L and S80F, respectively) were detected by PCR and sequencing (*1*). All 3 MRSA isolates were negative by PCR for the Panton-Valentine l**eukocidin,** toxic shock syndrome toxin 1, and exfoliative toxins A and B.

The patient lived with his wife and 2 sons near a pig farm. Both sons worked on the farm; the patient, but not his wife, helped sporadically on the farm. Nasal samples from the 3 family members indicated MRSA carriage in 1 son but not in the other son or the patient’s wife. The characteristics of the nasal MRSA strain recovered from the son were identical to those previously detected in MRSA strains from the patient (Table). In addition, nasal swabs from 18 pigs on the farm were randomly taken, and MRSA isolates were detected in 9 (50%) pigs; 1 MRSA isolate per animal was further characterized. Eight isolates were typed as ST398/t011/SCC*mec*V/*agr*I, and the remaining one as ST398/t1451/SCC*mec*V/*agr*I. All animal isolates had the same resistance phenotype and genotype as the MRSA isolates from the patient and son. None harbored the studied virulence factors (Table). All isolates had an unusual clindamycin-resistance/erythromycin-susceptibility phenotype and harbored the *vga*(A) gene.

We analyzed all MRSA isolates of human and animal origins by *Apa*I-PFGE (*2*) and compared patterns as previously recommended (*3*). Only 1 pulsotype (A) and 3 closely related subtypes were identified (A1, A2, and A3). One MRSA isolate obtained from pleural fluid of the patient, 2 isolates from nasal swabs (patient and son), and most isolates from animals showed the same PFGE pulsotype and subtype (A1). Alternatively, 1 MRSA isolate from bronchial aspirate of the patient and 2 isolates from animals showed closely related patterns (subtypes A2 and A3).

Other studies have suggested clonal spread and transmission of MRSA ST398 between pigs and persons who work with them (*4**,**5*). This microorganism has been generally associated with skin and soft tissue infections in humans (*6*). Nevertheless, severe infections by ST398 also have been sporadically described, and the report of 7 pneumonia cases associated with mechanical ventilation in central Europe is relevant (*7*). In general, ST398 isolates have fewer virulence factors than do other clones of MRSA (*2*); nonetheless, human infections from Panton-Valentine l**eukocidin–**positive ST398 isolates have been reported (*8*). The immunocompromised status of patients in intensive care units could favor dissemination of ST398 in this environment.

MRSA ST398 implicated in the described empyema was resistant to the first-line antimicrobial agent used for treatment (levofloxacin, MIC 4 mg/L) that was associated with amino acid changes in GyrA and ParC proteins, which could have accelerated the deteriorating evolution of the patient’s respiratory infection. The atypical clindamycin-resistance/erythromycin-susceptibility phenotype detected in our human and animal MRSA strains is infrequently detected in clinical MRSA isolates from humans. Nevertheless, this phenotype might be emerging among livestock MRSA isolates, as we and others (*9**,**10*) have observed. The *vga*(A) gene detected in these isolates could be responsible for this resistant phenotype, as has been recently reported by others (*10*).

In conclusion, we report potential pig-to-human transmission of MRSA ST398. MRSA ST398 can be associated with severe respiratory pathology in immunocompromised patients, and these microorganisms could also be resistant to other first-line antimicrobial agents, such as fluoroquinolones, used to treat these infections. Moreover, the unusual clindamycin-resistance/erythromycin-susceptibility phenotype might be a key marker (in addition to tetracycline resistance) for the possible presence of livestock-associated MRSA.

**Table:** Characteristics of methicillin-resistant *Staphylococcus aureus* strains recovered from humans and animals, Spain, 2009*

Strain	Origin	SCC*mec* type	MLST/*spa* type	*agr*	PFGE	Antimicrobial resistance phenotype	Resistance genes detected	Amino acid change	
								GrlA	GyrA
C2355	Patient, pleural fluid	V	ST398/t011	I	A1	OXA-CLI-TET-CIP-LEV	*mecA*, *tetK*, *tetM*, *vga*(A)	S80F	S84L
C2354	Patient, bronchial aspirate	V	ST398/t011	I	A2	OXA-CLI-TET-CIP-LEV	*mecA*, *tetK*, *tetM*, *vga*(A)	S80F	S84L
C2634	Patient, nasal swab	V	ST398/t011	I	A1	OXA-CLI-TET-CIP-LEV	*mecA*, *tetK*, *tetM*, *vga*(A)	S80F	S84L
C2664	Son, nasal swab	V	ST398/t011	I	A1	OXA-CLI-TET-CIP-LEV	*mecA*, *tetK*, *tetM*, *vga*(A)	S80F	S84L
C2669	Pig 1, nasal swab	V	ST398/t011	I	A1	OXA-CLI-TET-CIP-LEV	*mecA*, *tetK*, *tetM*, *vga*(A)	S80F	S84L
C2670	Pig 2, nasal swab	V	ST398/t011	I	A1	OXA-CLI-TET-CIP-LEV	*mecA*, *tetK*, *tetM*, *vga*(A)	S80F	S84L
C2694	Pig 3, nasal swab	V	ST398/t011	I	A1	OXA-CLI-TET-CIP-LEV	*mecA*, *tetK*, *tetM*, *vga*(A)	S80F	S84L
C2695	Pig 4, nasal swab	V	ST398/t011	I	A1	OXA-CLI-TET-CIP-LEV	*mecA*, *tetK*, *tetM*, *vga*(A)	S80F	S84L
C2697	Pig 5, nasal swab	V	ST398/t011	I	A3	OXA-CLI-TET-CIP-LEV	*mecA*, *tetK*, *tetM*, *vga*(A)	S80F	S84L
C2698	Pig 6, nasal swab	V	ST398/t011	I	A1	OXA-CLI-TET-CIP-LEV	*mecA*, *tetK*, *tetM*, *vga*(A)	S80F	S84L
C2700	Pig 7, nasal swab	V	ST398/t011	I	A3	OXA-CLI-TET-CIP-LEV	*mecA*, *tetK*, *tetM*, *vga*(A)	S80F	S84L
C2704	Pig 8, nasal swab	V	ST398/t011	I	A1	OXA-CLI-TET-CIP-LEV	*mecA*, *tetK*, *tetM*, *vga*(A)	S80F	S84L
C2706	Pig 9, nasal swab	V	ST398/t1451	I	A1	OXA-CLI-TET-CIP-LEV	*mecA*, *tetK*, *tetM*, *vga*(A)	S80F	S84L

*All strains were susceptible to fusidic acid, fosfomycin, gentamicin, tobramycin, mupirocin, trimethoprim-sulfamethoxazole, vancomycin, teicoplanin, quinupristin/dalfopristin, and tigecycline. SCC, staphylococcal cassette chromosome; MLST, multilocus sequence typing; PFGE, pulsed-field gel electrophoresis; ST, sequence type; OXA, oxacillin; CLI, clindamycin; TET, tetracycline; CIP, ciprofloxacin; LEV, levofloxacin.
